# Supplementary material for: Evolutionary ecology of microbial populations inhabiting deep sea sediments associated with cold seeps
Source: Nat Commun. 2023 Feb 28;14:1127. doi: 10.1038/s41467-023-36877-3 (PMC9974965; doi:10.1038/s41467-023-36877-3)
Supplement: Supplementary file 1 — Supplementary Information [file 41467_2023_36877_MOESM1_ESM.pdf]

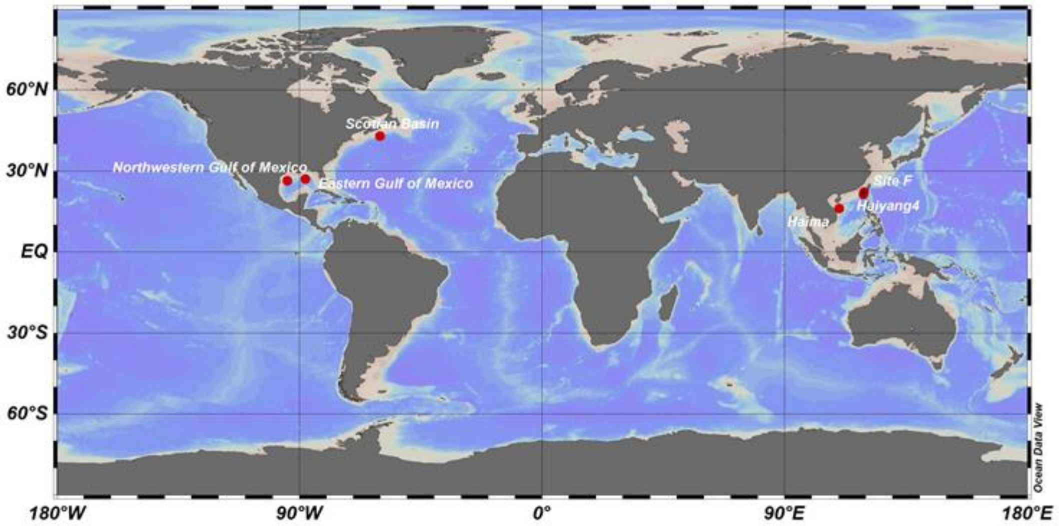

Supplementary Figure 1. Geographical distributions of six cold seep sites analyzed in this study. These sites were as follows: Eastern Gulf of Mexico; Northwestern Gulf of Mexico; Scotian Basin; Haiyang4, Site F, and Haima cold seeps in the South China Sea. Further details for each metagenome can be found in Supplementary Data 1. The world map was drawn using the Ocean Data View v5.4.0.

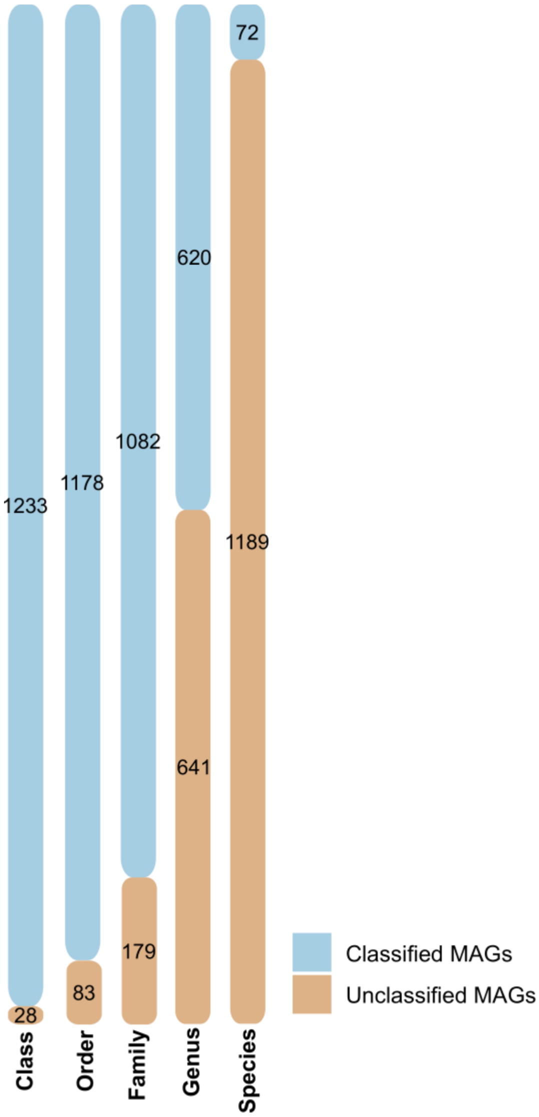

Supplementary Figure 2. The proportion of MAGs classified by GTDB-Tk at each taxonomic level. The proportion of MAGs is lower with increasing taxonomic resolution, with approximately half being able to be classified at the genus level. Detailed data for information of each MAG can be found in Supplementary Data 2.

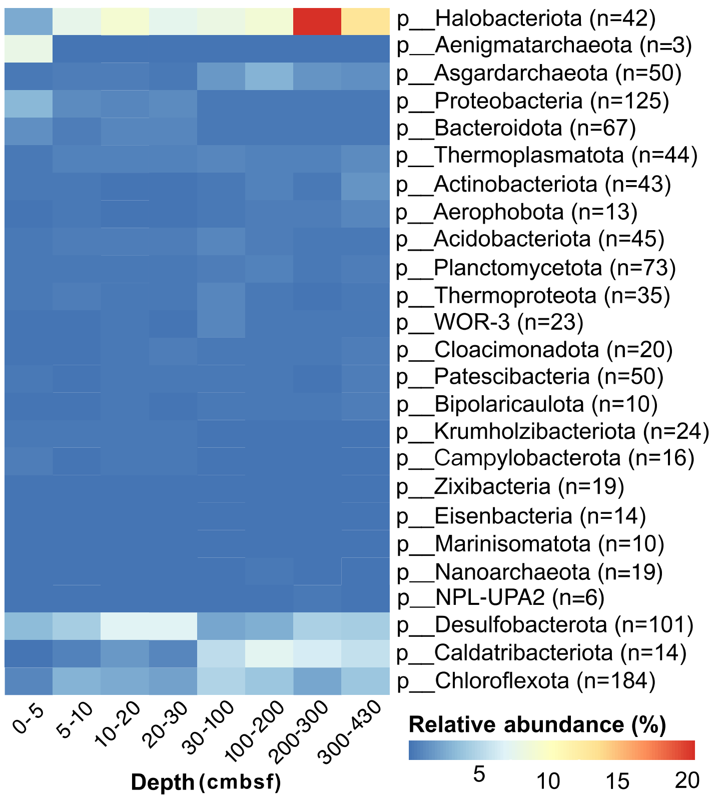

Supplementary Figure 3. Relative abundances of cold seep sediment microbial communities at phylum level. The 68 samples were catalogued into eight depth groups: 0-5 cmbsf (*n* = 11); 5-10 cmbsf (*n* = 14); 10-20 cmbsf (*n* = 16); 20-30 cmbsf (*n* = 8); 30-100 cmbsf (*n* = 6);100-200 cmbsf (*n* = 4); 200-300 cmbsf (*n* = 4); 300-430 cmbsf (*n* = 5). The reported total relative abundance of each phylum was averaged from depth groups. Detailed data for relative abundance of each population in each sample can be found in Supplementary Data 3.

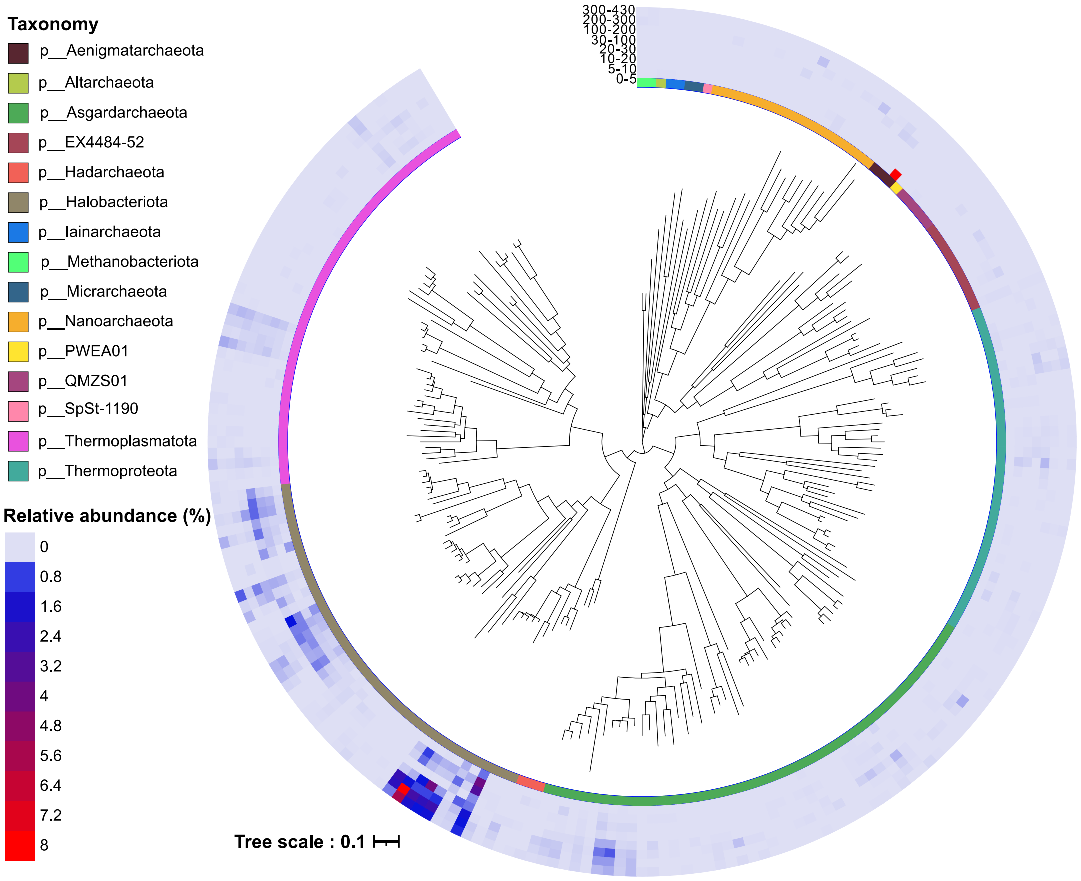

Supplementary Figure 4. Maximum likelihood phylogenetic tree of assembled archaeal MAGs with their relative abundance. The maximum-likelihood phylogenomic tree was built based on concatenated amino acid sequences of 122 archaeal marker genes produced by GTDB-Tk. Scale bar indicates the mean number of substitutions per site. See Supplementary Figure 3 and Supplementary Data 3 for more details about depth-profile relative abundances.

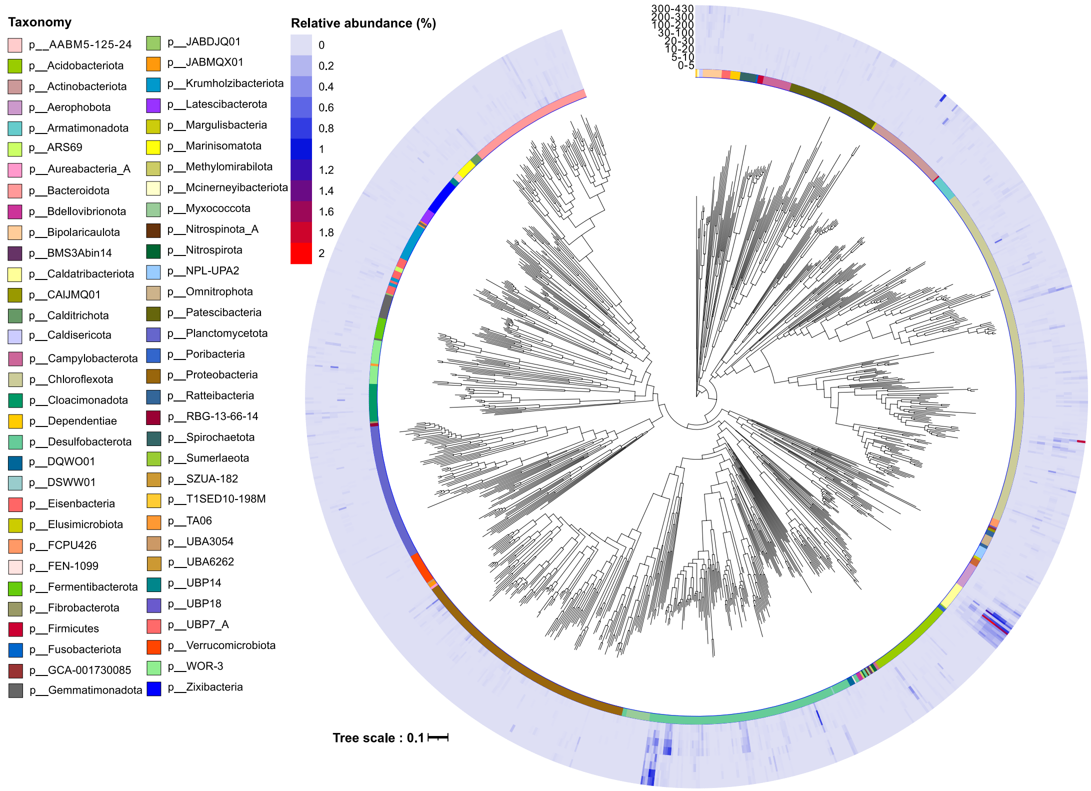

Supplementary Figure 5. Maximum likelihood phylogenetic tree of assembled bacterial MAGs with relative abundance. The maximum-likelihood phylogenomic tree was built based on concatenated amino acid sequences of 120 bacterial marker genes produced by GTDB-Tk. Scale bar indicates the mean number of substitutions per site. See Supplementary Figure 3 and Supplementary Data 3 for more details about depth-profile relative abundances.

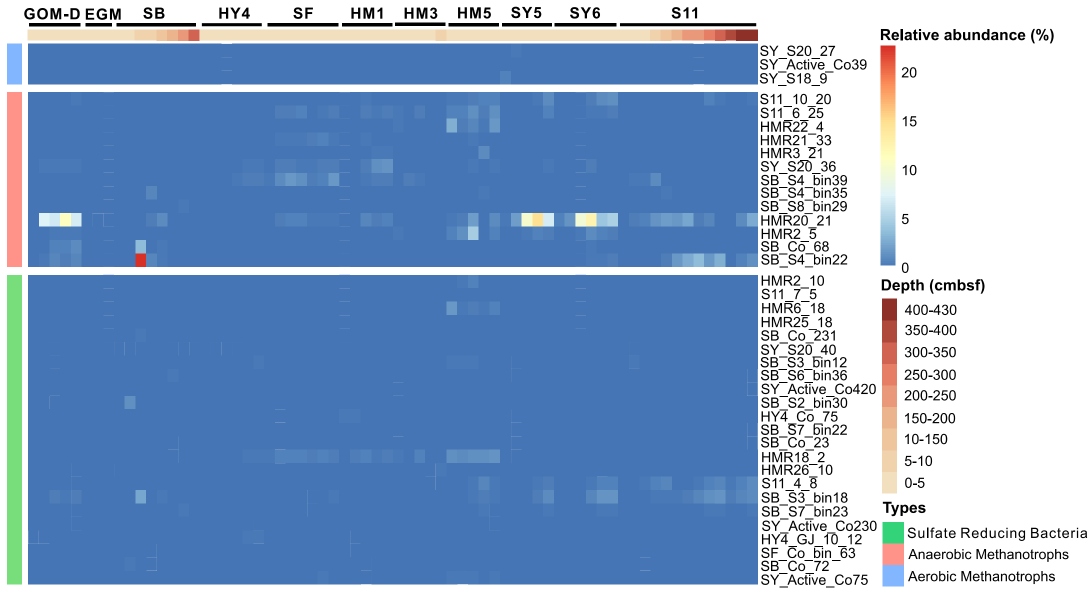

Supplementary Figure 6. Relative abundances of 39 selected microbial species used for microdiversity analysis. These species belong to aerobic methane-oxidizing bacteria, anaerobic methanotrophic archaea and sulfate-reducing bacteria, that are three key groups of functional microorganisms in cold seeps sediments. The x-axis represents every single sample in the study arranged by site, with the color scale indicating the depth interval of the sample. The y-axis displays MOB, ANME, SRB taxonomic groups. Fill color intensity reflects the relative abundance of a given genome at a given sample. Detailed data for relative abundance of each population in each sample can be found in Supplementary Data 3.

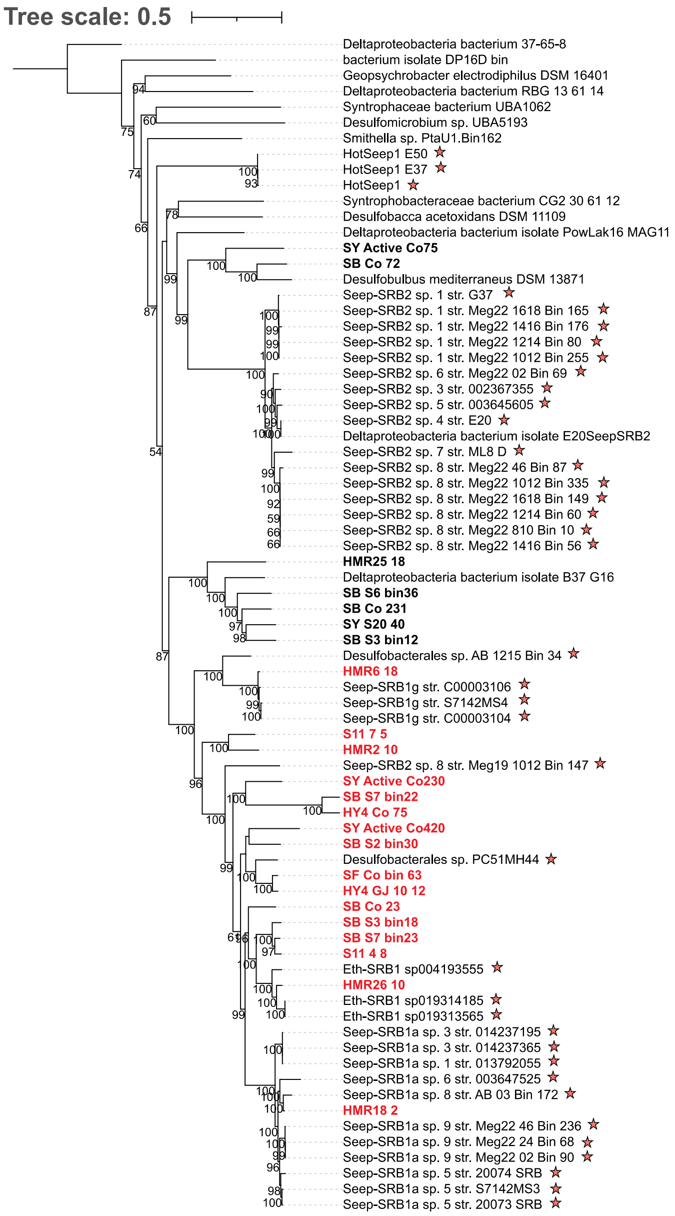

Supplementary Figure 7. Maximum-likelihood phylogenetic tree based on 120 single-copy marker genes in bacteria. 39 genomes from four syntrophic SRB clades (HotSeep-1, Seep-SRB2, Seep-SRB1a and Seep-SRB1g) collected from previous studies are highlighted with red stars (see Methods). Identified SRB genomes based on DsrA proteins from this study (n = 23) are in bold. SRB species cluster closely with the typical syntrophic SRB partners of ANME clades are labelled in red, referred to as syntrophic SRB. Black text at the nodes indicates bootstrap values. Scale bars indicate the average number of substitutions per site.

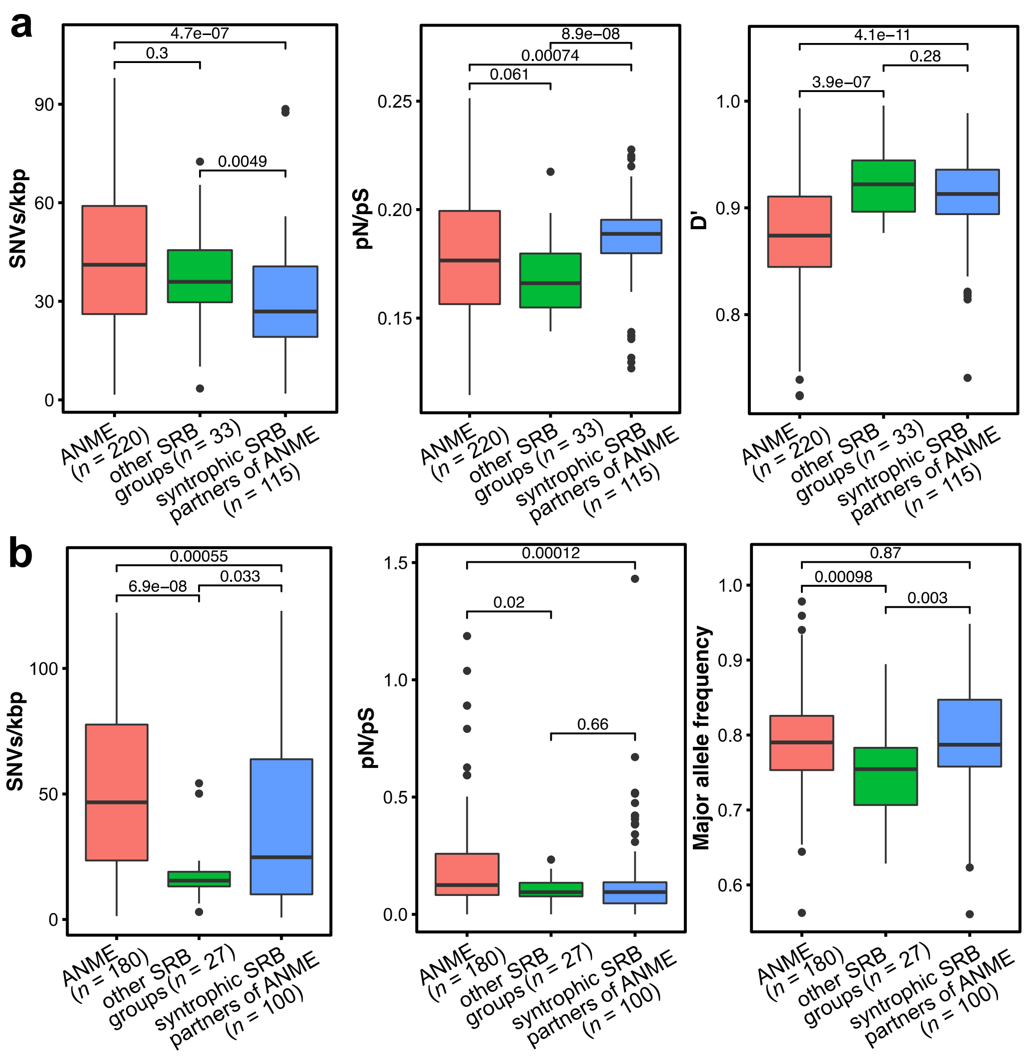

Supplementary Figure 8. Genome-wide and gene-wide comparison of evolutionary metrics for ANME, syntrophic SRB partners of ANME and other SRB groups in cold seep sediments. (a) Comparison of SNV density, D’ and pN/pS of genomes across ANME, syntrophic SRB partners of ANME and other SRB groups. (b) Comparison of SNV density, pN/pS and major allele frequency of *dsrA* and *mcrA* genes across ANME, syntrophic SRB partners of ANME and other SRB groups. P-values of differences across different groups were calculated using two-sided Wilcoxon test. Boxplot components: center lines, medians; box limits, 25th and 75th percentiles; whiskers, 1.5× interquartile range from the 25th and 75th percentiles; points, outliers. *n* values refer to the number of independent results used to derive statistics. Source data are provided as a Source Data file.

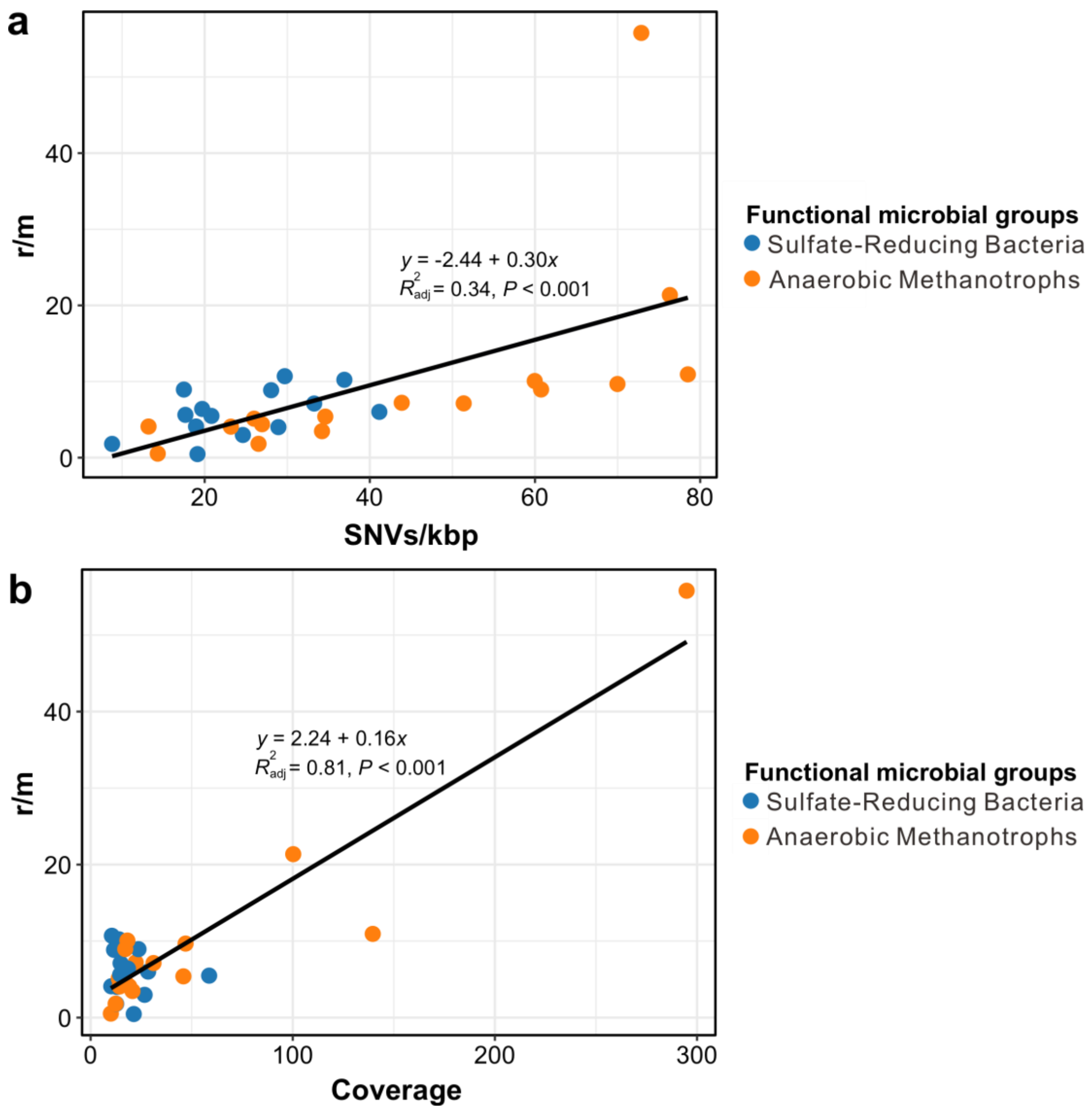

Supplementary Figure 9. Genome-wide comparison of evolutionary metrics for anaerobic methanotrophic archaea and sulfate-reducing bacteria in cold seep sediments. (a) Ratio of recombination to mutation (r/m) in relation to SNV density. (b) Ratio of r/m in relation to genome coverage. Each dot represents one species-level microbial population. Linear regressions and R^2 values are indicated for different taxonomic groups. Detailed statistics for linear regressions are provided in Supplementary Data 7.

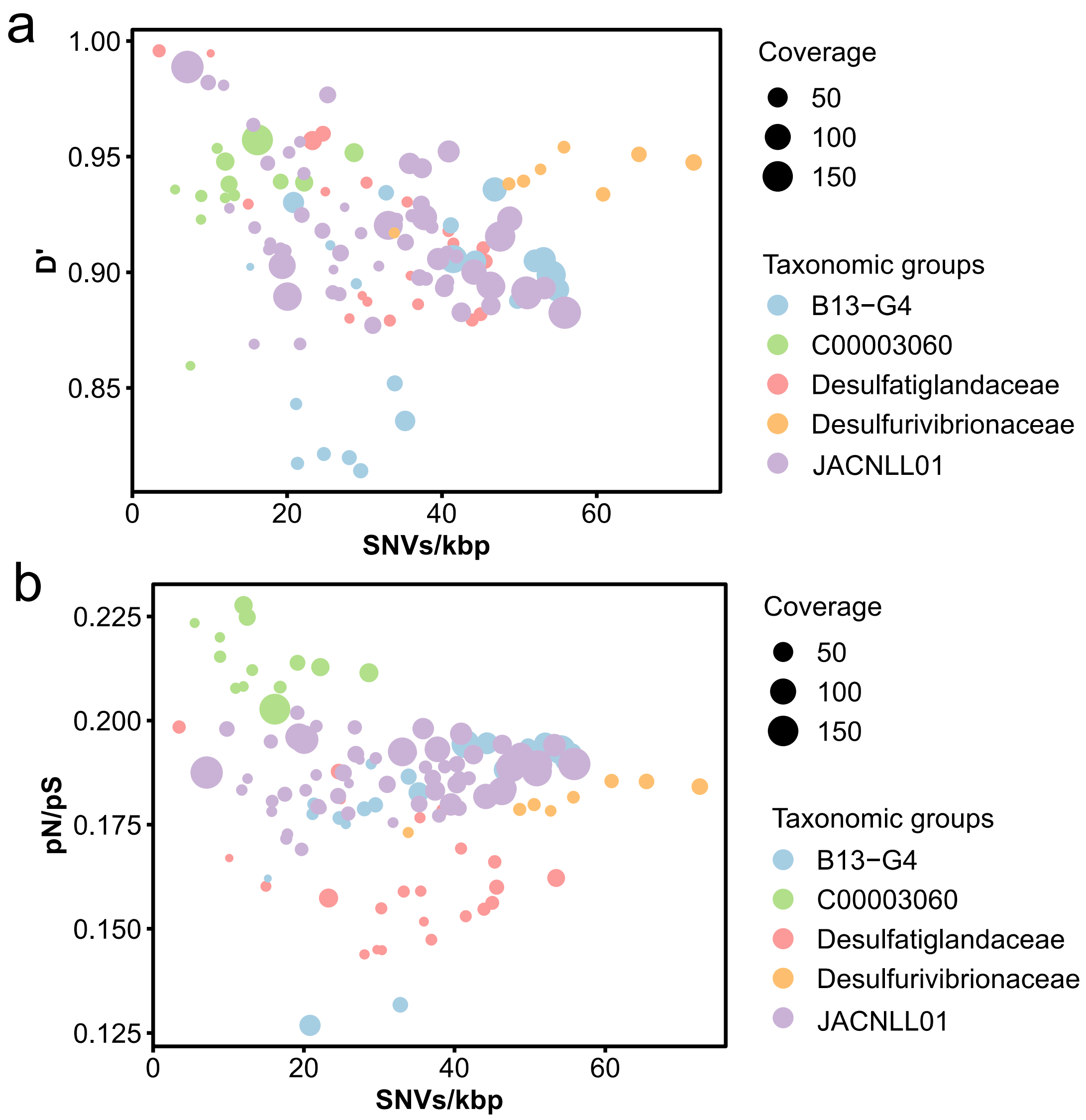

Supplementary Figure 10. Genome-wide evolutionary metrics of sulfate-reducing bacteria in cold seep sediments. (a) Relationships between SNV density, linkage disequilibrium (D’) and genome coverage. (b) Relationships between SNV density, pN/pS and genome coverage. Each dot represents one species-level microbial population. Source data are provided as a Source Data file.

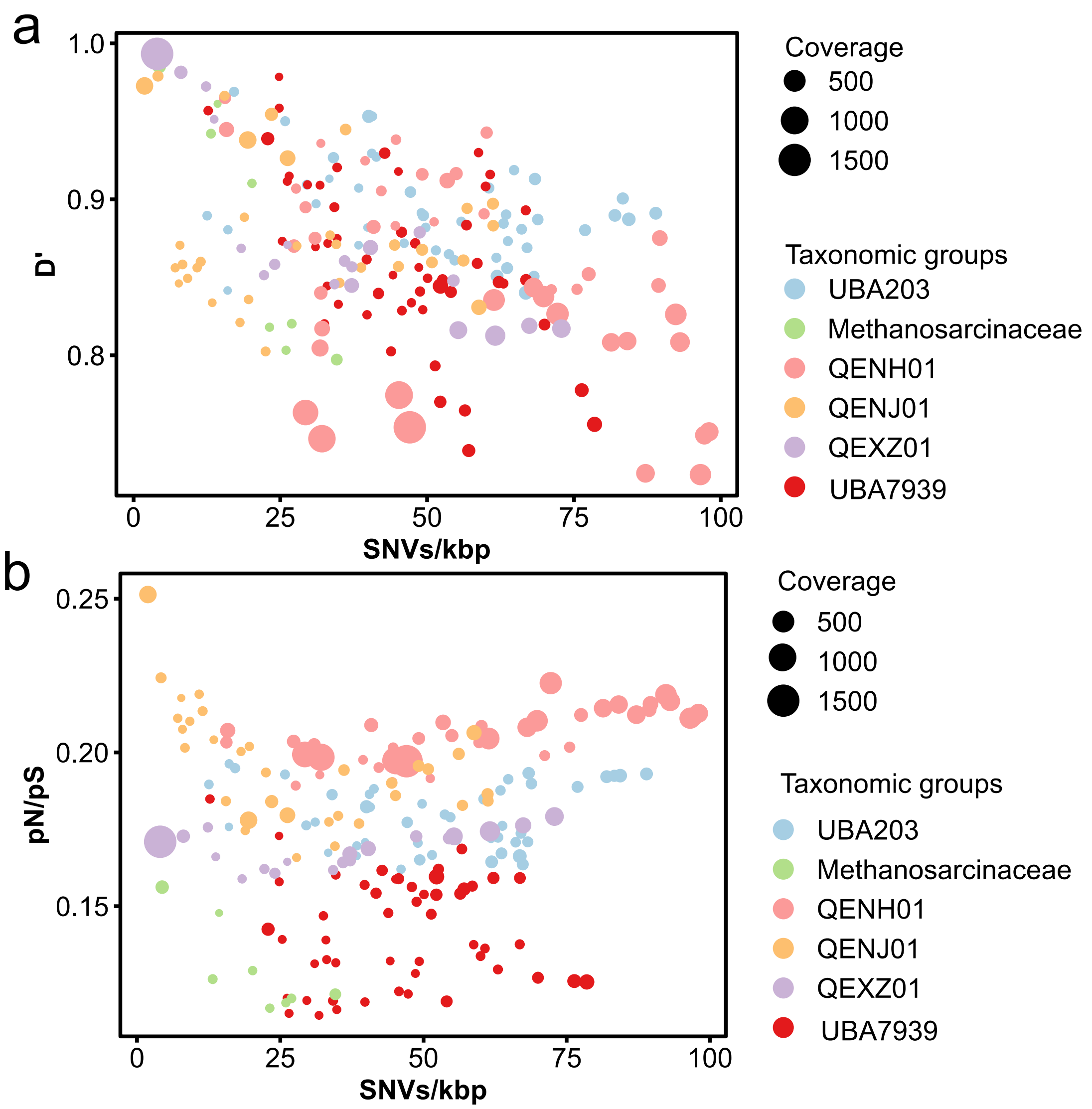

Supplementary Figure 11. Genome-wide evolutionary metrics of anaerobic methanotrophic archaea in cold seep sediments. (a) Relationships between SNV density, linkage disequilibrium (D') and genome coverage. (b) Relationships between SNV density, pN/pS and genome coverage. Each dot represents one species-level microbial population. Source data are provided as a Source Data file.

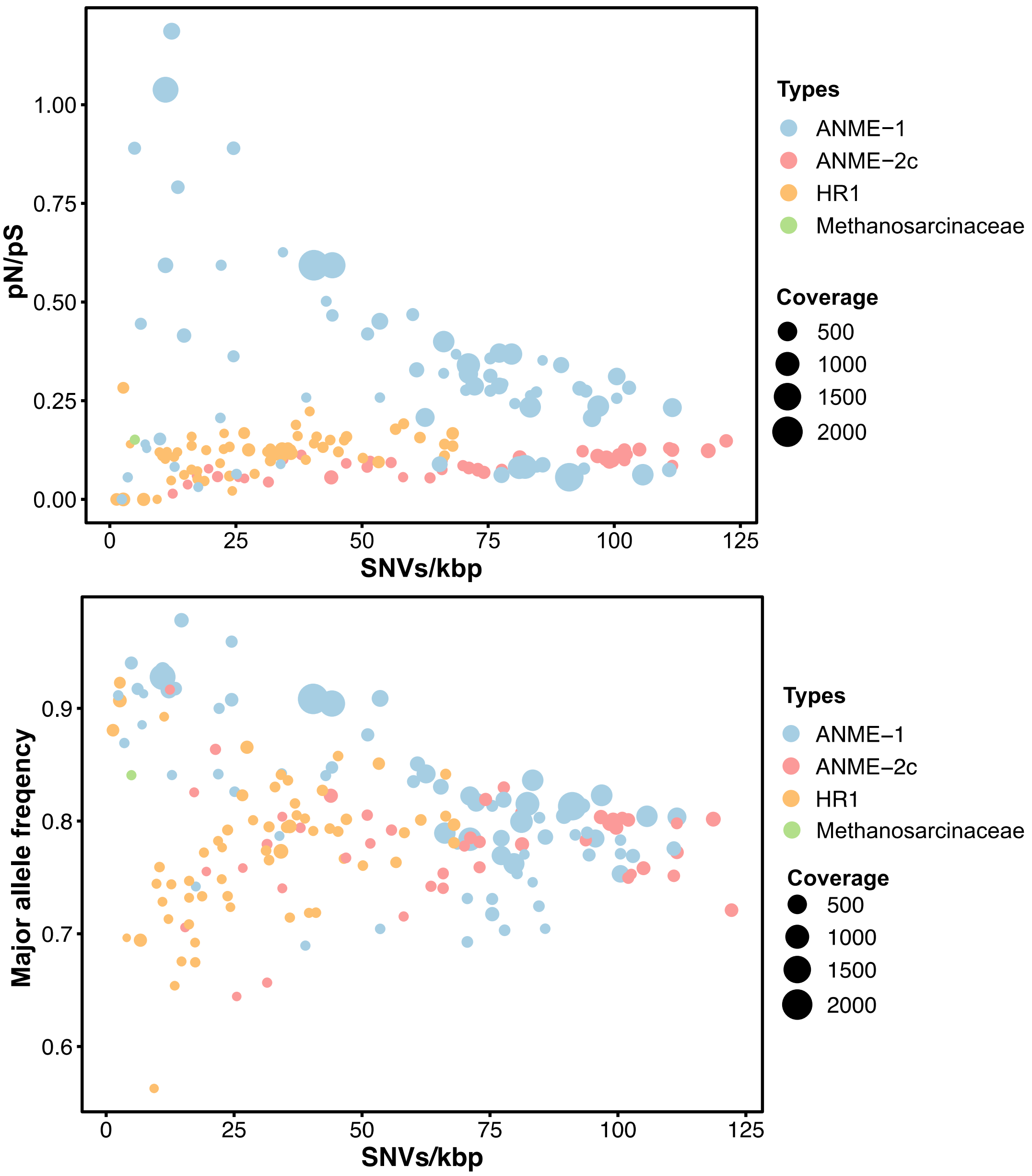

Supplementary Figure 12. Gene-specific evolutionary metrics of anaerobic methanotrophic archaea in cold seep sediments. (a) Relationships between SNV density, pN/pS and gene coverage at gene level. (b) Relationships between SNV density, major allele frequency and gene coverage at gene level. Each dot represents one species-level microbial population. Source data are provided as a Source Data file.

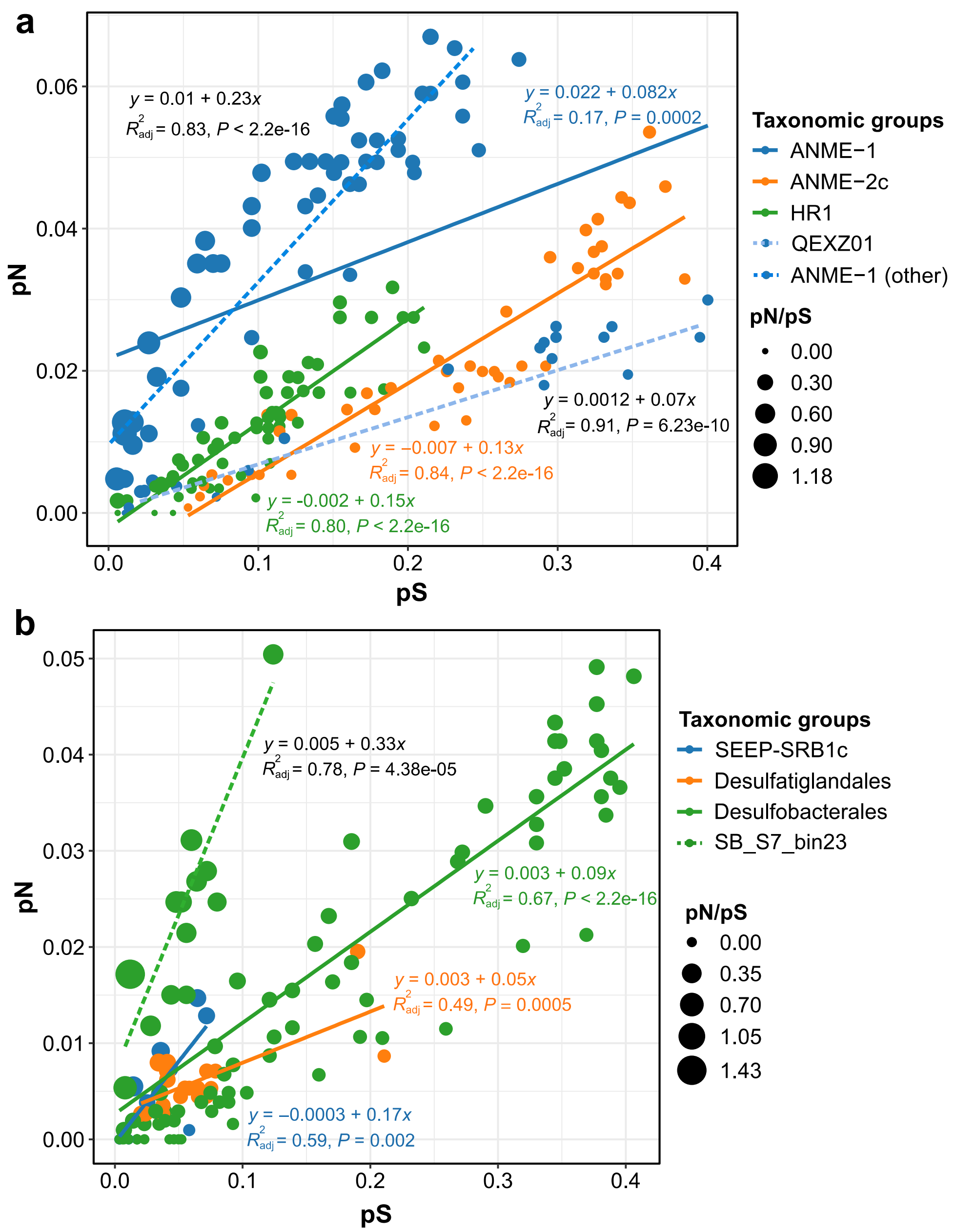

Supplementary Figure 13. Comparison between pN and pS of functional genes in cold seep sediments. (a) Linear regressions for *mcrA* genes from different taxonomic groups. ANME-1 (other) represents the taxonomic groups except for QEXZ01 in ANME-1. (b) Linear regressions for *dsrA* genes from different taxonomic group. Detailed statistics for linear regressions are provided in Supplementary Data 6. Source data are provided as a Source Data file.

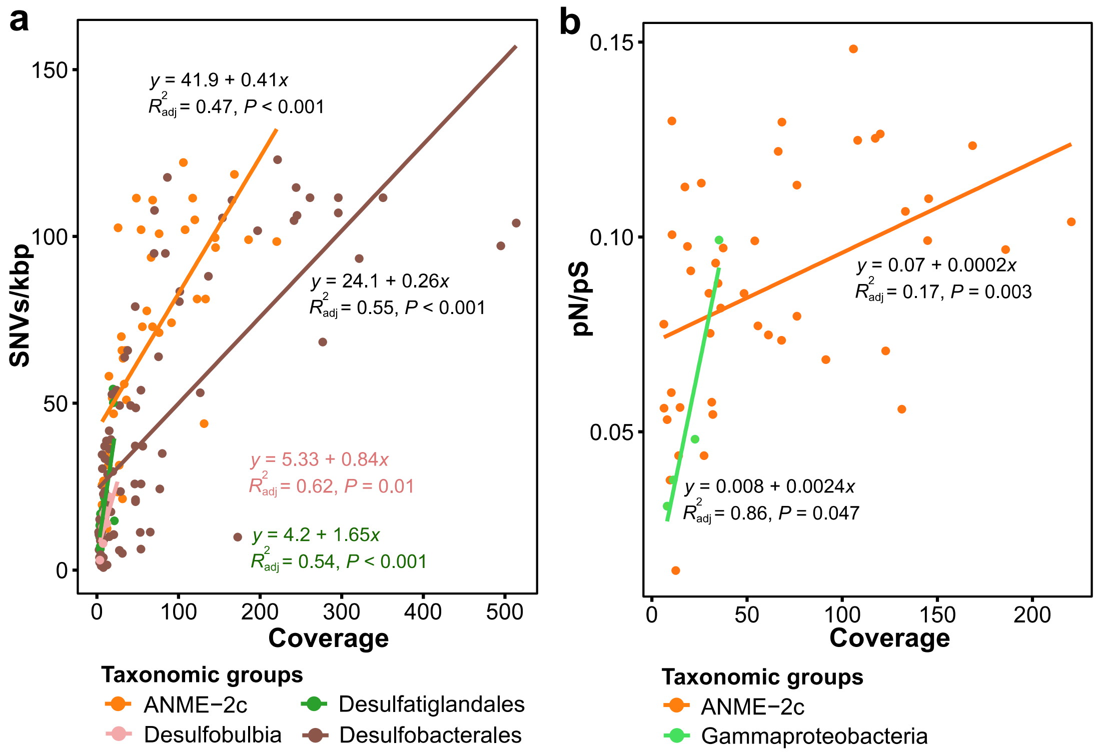

Supplementary Figure 14. Comparison of evolutionary metrics for microbial populations in cold seep sediments at the gene level. (a) SNV density in relation to gene coverage. (a) pN/pS in relation to gene coverage. Each dot represents one species-level microbial population. Linear regressions and R^2 values are indicated for different taxonomic groups. Detailed statistics for linear regressions are provided in Supplementary Data 6.

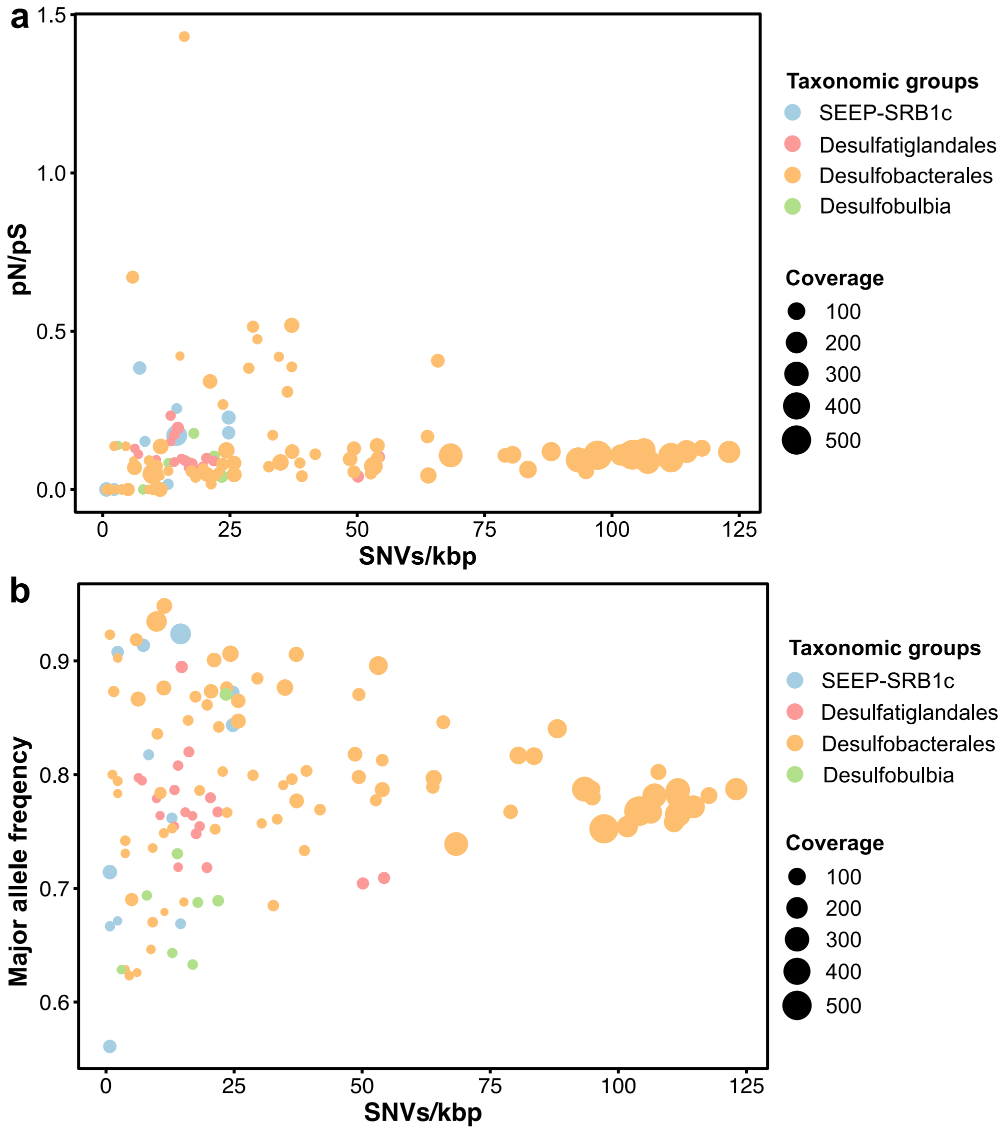

Supplementary Figure 15. Gene-specific evolutionary metrics of sulfate-reducing bacteria in cold seep sediments. (a) Relationships between SNV density, pN/pS and gene coverage at gene level. (b) Relationships between SNV density, major allele frequency and gene coverage at gene level. Each dot represents one species-level microbial population. Source data are provided as a Source Data file.

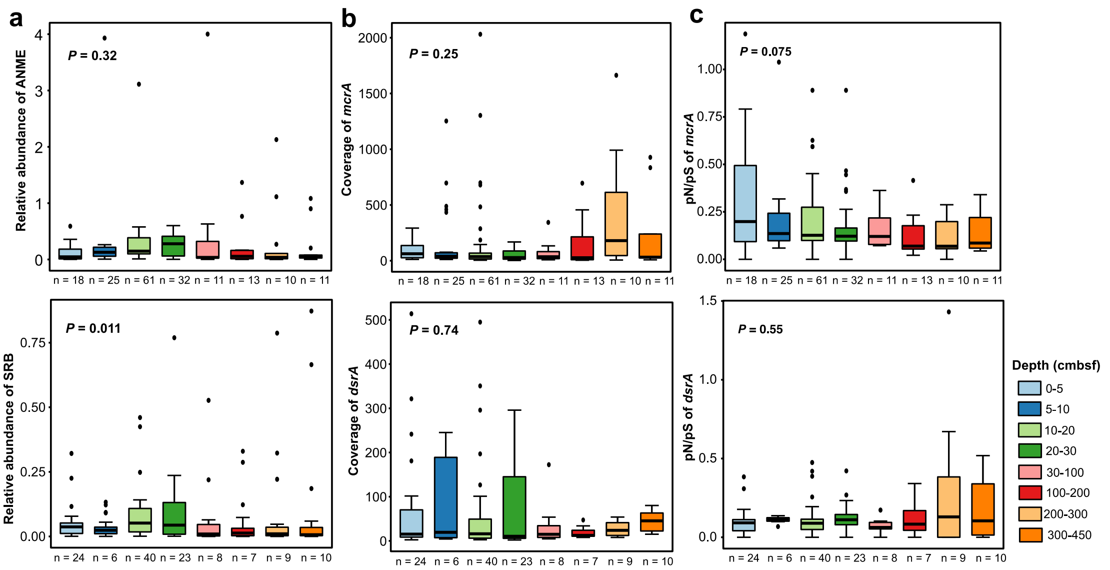

Supplementary Figure 16. Depth-specific comparison of relative abundance and evolutionary metrics for three key functional microbial groups and genes in cold seep sediments. (a) Box plot showing relative abundance of ANME and SRB genomes across eight depth groups. (b) Box plot showing gene coverage of *dsrA* and *mcrA* genes across eight depth groups. (b) Box plot showing pN/pS of *dsrA* and *mcrA* genes across eight depth groups. P-values of differences across different taxonomic groups were calculated using Kruskal-Wallis Rank Sum test. Boxplot components: center lines, medians; box limits, 25th and 75th percentiles; whiskers, 1.5× interquartile range from the 25th and 75th percentiles; points, outliers. *n* values refer to the number of independent results used to derive statistics. Detailed data for relative abundance of population can be found in Supplementary Data 3. Source data are provided as a Source Data file.

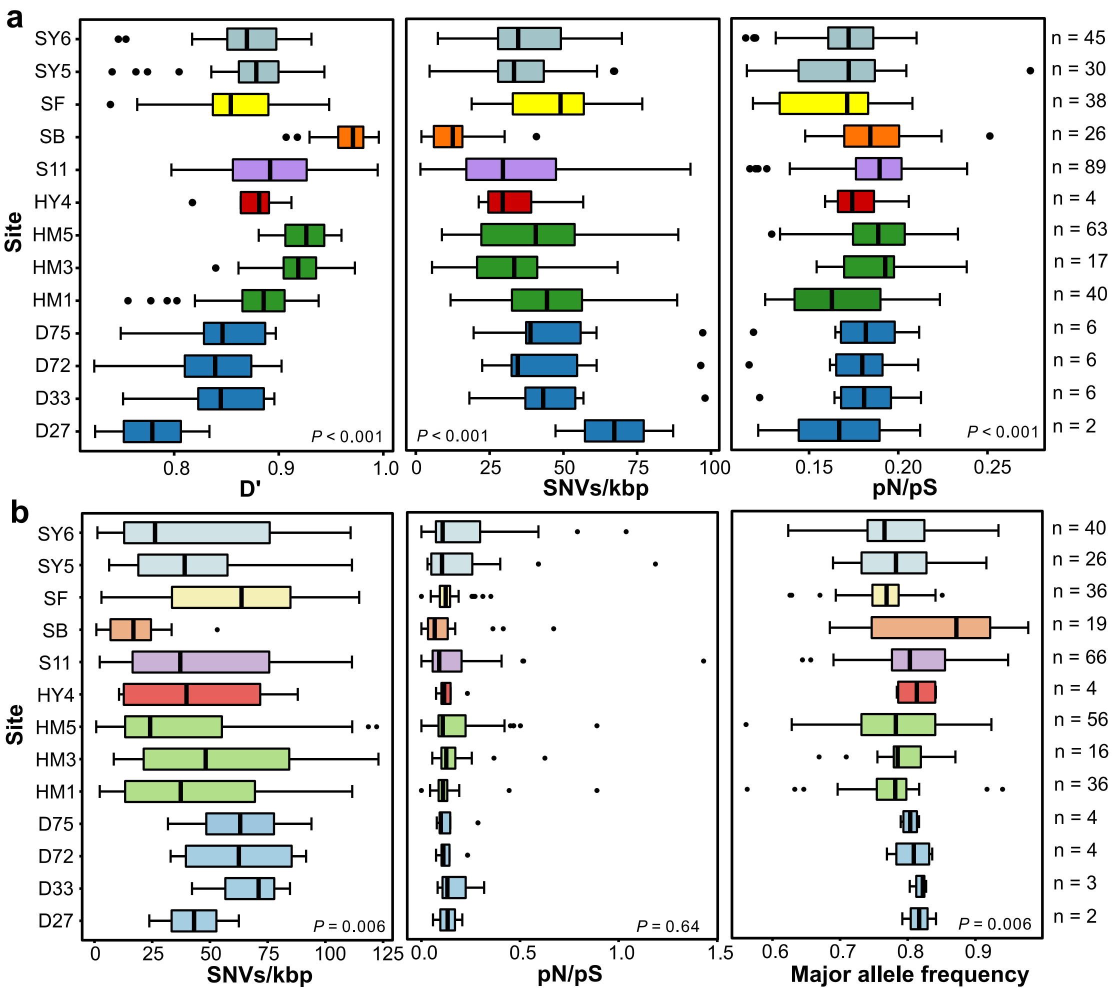

Supplementary Figure 17. Site-specific comparison of evolutionary metrics for three key functional microbial groups in cold seep sediments. (a) Box plot showing D', SNV density and pN/pS ratio at genome level across different cold seep sites. (b) Box plot showing SNV density, pN/pS and major allele frequency of key functional genes (*pmoA*, *dsrA* and *mcrA*) across different cold seep sites. P-values of differences across different taxonomic groups were calculated using Kruskal-Wallis Rank Sum test. Boxplot components: center lines, medians; box limits, 25th and 75th percentiles; whiskers, 1.5× interquartile range from the 25th and 75th percentiles; points, outliers. *n* values refer to the number of independent results used to derive statistics. Source data are provided as a Source Data file. Detailed statistics for significance tests are provided in **Supplementary Data 11**.

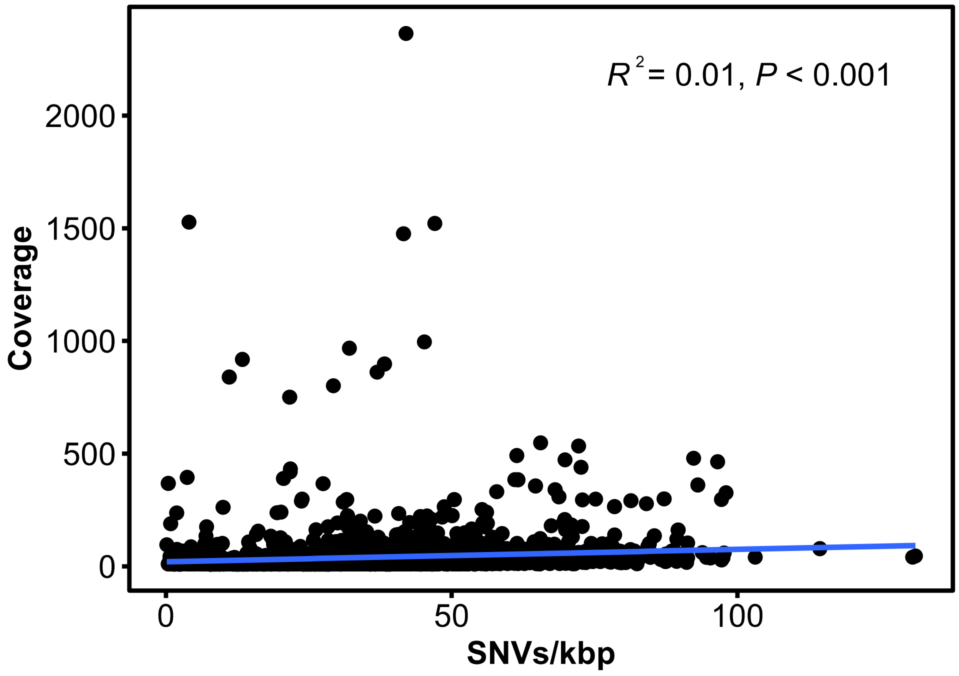

Supplementary Figure 18. Relationship between SNVs/kbp and coverage determined by two-tailed Pearson correlation test. Each symbol represents one species-level microbial population.
