# Supplementary material for: Evolutionary ecology of microbial populations inhabiting deep sea sediments associated with cold seeps
Source: Nat Commun. 2023 Feb 28;14:1127. doi: 10.1038/s41467-023-36877-3 (PMC9974965; doi:10.1038/s41467-023-36877-3)
Supplement: Supplementary file 3 — Description of Additional Supplementary Files [file 41467_2023_36877_MOESM3_ESM.docx]

**Description of Additional Supplementary Files**

**File Name:** Supplementary Data 1

**Description:** Sampling and geographic information about each site.

**File Name:** Supplementary Data 2

**Description:** Summary statistics of archaeal and bacterial MAGs assembled from cold seep samples.

**File Name:** Supplementary Data 3

**Description:** Relative abundance of archaeal and bacterial populations analyzed for microbial evolution in each sample.

**File Name:** Supplementary Data 4

**Description:** Functional annotation results based on METABOLIC for 1261 MAGs.

**File Name:** Supplementary Data 5

**Description:** Taxonomic information of MAGs used for Figures 4 and 6.

**File Name:** Supplementary Data 6

**Description:** Summary statistics of correlation analysis, linear fitting and significance tests among genetic variation indexes.

**File Name:** Supplementary Data 7

**Description:** Summary statistics of r/m values in genomes and linear fitting among genetic variation indexes.

**File Name:** Supplementary Data 8

**Description:** Summary of evolutionary metrics and bacterial and archaeal numbers estimated by qPCR of 16S rRNA genes from the SB site.

**File Name:** Supplementary Data 9

**Description:** Summary statistics of correlation analysis and linear fitting between genetic variation indexes and depth.

**File Name:** Supplementary Data 10

**Description:** Summary statistics of two-tailed pearson correlation analysis of geochemical parameters and evolutionary metrics.

**File Name:** Supplementary Data 11

**Description:** Summary statistics of significance tests of genetic variation among sites and depth groups.
