# Supplementary material for: Evolutionary ecology of microbial populations inhabiting deep sea sediments associated with cold seeps
Source: Nat Commun. 2023 Feb 28;14:1127. doi: 10.1038/s41467-023-36877-3 (PMC9974965; doi:10.1038/s41467-023-36877-3)
Supplement: Supplementary file 15 — Reporting Summary [file 41467_2023_36877_MOESM15_ESM.pdf]

nature portfolio

Corresponding author(s): Xiyang Dong

Last updated by author(s): Feb 12, 2023

Reporting Summary

Nature Portfolio wishes to improve the reproducibility of the work that we publish. This form provides structure for consistency and transparency in reporting. For further information on Nature Portfolio policies, see our Editorial Policies and the Editorial Policy Checklist.

Statistics

For all statistical analyses, confirm that the following items are present in the figure legend, table legend, main text, or Methods section.

n/a	Confirmed	
☐	☒	The exact sample size (*n*) for each experimental group/condition, given as a discrete number and unit of measurement
☒	☐	A statement on whether measurements were taken from distinct samples or whether the same sample was measured repeatedly
☐	☒	The statistical test(s) used AND whether they are one- or two-sided<br>*Only common tests should be described solely by name; describe more complex techniques in the Methods section.*
☒	☐	A description of all covariates tested
☐	☒	A description of any assumptions or corrections, such as tests of normality and adjustment for multiple comparisons
☐	☒	A full description of the statistical parameters including central tendency (e.g. means) or other basic estimates (e.g. regression coefficient) AND variation (e.g. standard deviation) or associated estimates of uncertainty (e.g. confidence intervals)
☐	☒	For null hypothesis testing, the test statistic (e.g. *F*, *t*, *r*) with confidence intervals, effect sizes, degrees of freedom and *P* value noted<br>*Give P values as exact values whenever suitable.*
☒	☐	For Bayesian analysis, information on the choice of priors and Markov chain Monte Carlo settings
☒	☐	For hierarchical and complex designs, identification of the appropriate level for tests and full reporting of outcomes
☐	☒	Estimates of effect sizes (e.g. Cohen's *d*, Pearson's *r*), indicating how they were calculated

Our web collection on statistics for biologists contains articles on many of the points above.

Software and code

Policy information about availability of computer code

Data collection	No software was used for data collection. All raw sequencing reads were collected from published literatures manually or reused from our previous studies.
Data analysis	Metagenomic raw reads were checked for quality, assembled and binned using the metaWRAP v1.3.2 pipeline. Filtered reads from each metagenome were individually assembled and co-assembled using MEGAHIT v1.1.3. The quality of the obtained MAGs was estimated by the lineage-specific workflow of CheckM v1.0.12. All bins were dereplicated using dRep v3.2.2. Taxonomy assignment was performed using GTDB-TK v1.5.1. To calculate the relative abundance of each MAG, CoverM v0.6.0 was used in genome mode. METABOLIC v4.0 was used to predict metabolic and biogeochemical functional trait profiles of MAGs. The predicted genes were screened against custom protein databases using DIAMOND v0.9.14. For phylogenetic analysis, amino acid sequences were aligned using the MUSCLE algorithm included in the MEGA X v10.2.4. For phylogenetic analysis of typical syntrophic SRB partners of ANME, a concatenated alignment of 120 single-copy marker genes in bacteria was produced using GTDB-Tk v1.5.1. A maximum likelihood tree was constructed using IQ-TREE v2.0.5. All produced trees were visualized and beautified in the iTOL v6. Filtered reads were mapped to concatenated MAGs using Bowtie2 v2.2.5. Population statistics and nucleotide metrics including D', SNVs/kbp, pN/pS and major allele frequency were calculated using inStrain v1.5.4. Gene annotations of MAGs were performed with Prodigal v2.6.3 for the gene module of inStrain. The mcorr package (Jan 2022, https://github.com/kussell-lab/mcorr) was used to calculate the gamma/mu for each population. Statistical analysis was carried out in R v4.0.0.

For manuscripts utilizing custom algorithms or software that are central to the research but not yet described in published literature, software must be made available to editors and reviewers. We strongly encourage code deposition in a community repository (e.g. GitHub). See the Nature Portfolio guidelines for submitting code & software for further information.

Data

Policy information about availability of data

All manuscripts must include a data availability statement. This statement should provide the following information, where applicable:

- Accession codes, unique identifiers, or web links for publicly available datasets
- A description of any restrictions on data availability
- For clinical datasets or third party data, please ensure that the statement adheres to our policy

MAGs, files for the phylogenetic trees and other related information have been uploaded to Figshare (https://doi.org/10.6084/m9.figshare.17195003.v2). MAGs used for the evolutionary analysis have also been deposited in NCBI under BioProject ID PRJNA831433 (https://www.ncbi.nlm.nih.gov/bioproject/PRJNA831433). The databases used in this study include GTDB database R06-RS202 (https://data.gtdb.ecogenomic.org/releases/release202/), KEGG database (http://www.genome.ad.jp/kegg/), TIGRfam (https://tigrfams.jcvi.org/cgi-bin/index.cgi), Pfam (https://www.ebi.ac.uk/interpro/entry/pfam/), custom hidden Markov model (HMM) databases (https://github.com/banfieldlab/metabolic-hmms), dbCAN_seq (https://bcb.unl.edu/dbCAN_seq/), MEROPS (http://merops.sanger.ac.uk/) and the custom protein databases of representative PmoA, McrA and DsrA sequences (https://doi.org/10.26180/c.5230745). Source data are provided with this paper.

Field-specific reporting

Please select the one below that is the best fit for your research. If you are not sure, read the appropriate sections before making your selection.

☐ Life sciences ☐ Behavioural & social sciences ☒ Ecological, evolutionary & environmental sciences

For a reference copy of the document with all sections, see nature.com/documents/nr-reporting-summary-flat.pdf

Ecological, evolutionary & environmental sciences study design

All studies must disclose on these points even when the disclosure is negative.

Study description	To gain insights into evolutionary trajectories among microbial populations inhabiting cold seep sediments, we examined the metagenomic data of 68 cold seep sediment samples to track population microdiversity from metagenomic short-read alignments and performed microdiversity-aware genomic comparisons.
Research sample	Metagenomic data sets were compiled from 68 sediment samples (0 to 430 cmbsf) collected from six globally distributed cold seep sites. We chose them because there were only 68 metagenomic datasets publicly available and fulfilling the requirements for microdiversity analyses when we performed this study. These sites are as follows: Eastern Gulf of Mexico (EGM); Northwestern Gulf of Mexico (GOM-D); Scotian Basin (SB); Haiyang4 (HY4), Site F (SF), and Haima cold seeps in the South China Sea (HM1, HM3, HM5, SY5, SY6, S11). They represent six globally distributed areas of hydrocarbon seepage. Samples originate from two types of cold seeps, namely oil and gas seeps and methane seeps. For samples from Northwestern Gulf of Mexico, metagenomic data sets along with metadata were downloaded from NCBI Sequencing Read Archive (SRA) and NCBI BioProject databases (https://www.ncbi.nlm.nih.gov/bioproject/PRJNA553005). The other 63 metagenomic datasets used in this study were obtained from our previous publications, including a Scotian Basin cold seep in the northwest Atlantic Ocean (https://doi.org/10.1038/s41467-020-19648-2), an Eastern Gulf of Mexico cold seep (https://doi.org/10.1038/s41467-019-09747-0), and the South China Sea cold seeps Haiyang4 (https://doi.org/10.1111/1462-2920.15796), Site F (https://doi.org/10.1111/1462-2920.15796) and Haima (https://doi.org/10.1016/j.dsr.2021.103489, https://www.researchsquare.com/article/rs-2323106/v1, https://doi.org/10.1101/2022.12.21.518016). Geochemical parameters from SY and S11 sites and cell densities from the SB site were collected from our previous publications (https://www.researchsquare.com/article/rs-2323106/v1, https://doi.org/10.1101/2022.12.21.518016, https://doi.org/10.1038/s41467-020-19648-2).
Sampling strategy	No field sampling were performed in this study and metagenomic datasets were collected online or in house. For samples from Northwestern Gulf of Mexico, metagenomic data sets along with metadata were downloaded from NCBI Sequencing Read Archive (SRA) and NCBI BioProject databases (https://www.ncbi.nlm.nih.gov/bioproject/PRJNA553005). The other 63 metagenomic datasets used in this study were obtained from our previous publications, including a Scotian Basin cold seep in the northwest Atlantic Ocean (https://doi.org/10.1038/s41467-020-19648-2), an Eastern Gulf of Mexico cold seep (https://doi.org/10.1038/s41467-019-09747-0), and the South China Sea cold seeps Haiyang4 (https://doi.org/10.1111/1462-2920.15796), Site F (https://doi.org/10.1111/1462-2920.15796) and Haima (https://doi.org/10.1016/j.dsr.2021.103489, https://www.researchsquare.com/article/rs-2323106/v1, https://doi.org/10.1101/2022.12.21.518016). No statistical methods were used to predetermine sample size, as there were only 68 metagenomic datasets publicly available and fulfilling the requirements for microdiversity analyses when we performed this study.
Data collection	Metagenomic datasets from cold seep sites were publicly available or derived from our previous published studies, which were collected by the co-author Yong Wang, Xi Xiao, Jiwei Li, Casey R.J. Hubert. Metagenomic and evolutionary analyses were performed by the co-author Xiyang Dong and Yongyi Peng.
Timing and spatial scale	Metagenomic datasets from cold seep sites were collected from April 2019 to Dec 2020.
Data exclusions	No data was excluded.
Reproducibility	All analyses were computational and it's straightforward to reproduce the findings according to the described methods.
Randomization	Randomization is not relevant since our study aims to discover evolutionary trajectories of key bacteria and archaea in deep sea cold

	seep extreme environments. It is necessary to calculate and keep evolutionary indexes of all species-cluster representatives, using all metagenomic data collected from cold seep sites.
Blinding	Blinding was not necessary for the development of this study as it is mostly descriptive and treats environmental sequencing samples that cannot be influenced by human manipulation.

Did the study involve field work? ☐ Yes ☒ No

Reporting for specific materials, systems and methods

We require information from authors about some types of materials, experimental systems and methods used in many studies. Here, indicate whether each material, system or method listed is relevant to your study. If you are not sure if a list item applies to your research, read the appropriate section before selecting a response.

Materials & experimental systems

n/a	Involved in the study	
☒	☐	Antibodies
☒	☐	Eukaryotic cell lines
☒	☐	Palaeontology and archaeology
☒	☐	Animals and other organisms
☒	☐	Human research participants
☒	☐	Clinical data
☒	☐	Dual use research of concern

Methods

n/a	Involved in the study	
☒	☐	ChIP-seq
☒	☐	Flow cytometry
☒	☐	MRI-based neuroimaging
